# Biofilm Disruption Enhances Antimicrobial Therapy for Small Intestinal Bacterial Overgrowth and Intestinal Methanogen Overgrowth

**DOI:** 10.7759/cureus.99116

**Published:** 2025-12-13

**Authors:** Michael Ruscio, Gavin Guard, Darla O'Dwyer, Ray Darville, Hannah Klopf, Robert Abbott, Scott Spiridigliozzi

**Affiliations:** 1 Integrative Medicine, Ruscio Institute for Functional Health, Austin, USA; 2 Medicine, College of Health Sciences, University of Bridgeport, Bridgeport, USA; 3 Human Sciences, Stephen F. Austin State University, Nacogdoches, USA; 4 Anthropology, Geography and Sociology, Stephen F. Austin State University, Nacogdoches, USA; 5 Osteopathic Medicine, Ruscio Institute for Functional Health, Austin, USA

**Keywords:** anti-biofilm, dysbiosis, gut microbiota, herbal therapies, intestinal methanogen overgrowth, sibo, small intestinal bacterial overgrowth

## Abstract

Background

Small intestinal bacterial overgrowth (SIBO) and intestinal methanogen overgrowth (IMO) are forms of microbial dysbiosis linked to gastrointestinal as well as systemic symptoms and sequelae. Some patients prefer non-antibiotic treatment options, and thus, herbal antimicrobials are emerging as alternatives to rifaximin. Treatment with pharmaceuticals or herbals can be hindered via biofilm-related resistance. This retrospective chart review evaluated whether adding a biofilm disruptor to herbal antimicrobials enhanced SIBO/IMO eradication, as defined by standardized breath test criteria.

Methods

Thirteen patients with SIBO, IMO, or both, as determined by lactulose breath testing, had previously been randomized in a clinical protocol to receive either herbal antimicrobials alone (n = 5, control group) or herbal antimicrobials combined with biofilm disruptors (n = 8, treatment group) over an 8-week period.

Results

Hydrogen levels declined more in the treatment group compared with controls (−30.75 vs −11.40 ppm, within-group P = 0.007). Methane levels also decreased more in the treatment group than in controls (−26.38 vs −2.00 ppm, within-group P = 0.042). The SIBO eradication rate ranged from 60-100%, but did not differ significantly across groups. Neither group achieved eradication of IMO.

Conclusions

Adding a biofilm disruptor to herbal antimicrobials was associated with significantly greater reductions in hydrogen and methane gas levels. While SIBO eradication rates with herbal protocols - both with and without biofilm agents - were comparable to those seen with rifaximin, the differences were not statistically significant; importantly, this was likely due to the small sample size. Larger prospective, controlled studies are needed to validate these findings and clarify the potential role of biofilm disruptors in the management of SIBO/IMO.

## Introduction

Two now-distinct types of pathological bacterial fermentation, small intestinal bacterial overgrowth (SIBO) and intestinal methanogen overgrowth (IMO), are lab findings that fall under the umbrella of microbial dysbiosis. Though both types once fell under the term SIBO, current terminology separates SIBO and IMO by the dominant gases each produces: SIBO is hydrogen-dominant, and IMO is methane-dominant [1]. 

A bacterial concentration of >10^3^ colony-forming units/mL of jejunal aspirate is the gold standard for diagnosing SIBO/IMO [1]. Because endoscopic aspirate testing is not routine in clinical practice, consensus guidelines recommend breath testing with lactulose or glucose in patients showing symptoms of SIBO/IMO -such as flatulence, bloating, distension, diarrhea, constipation, or abdominal pain - to identify clinically relevant cases [1]. Given the variability in clinical presentation and reliance on breath testing for diagnosis, researchers have yet to determine the true prevalence of SIBO/IMO in the general population. However, available evidence suggests that incidence increases with age [2,3]. 

Clinically, SIBO/IMO may lead to maldigestion, malabsorption, and altered motility [4-6], and may be implicated in the pathogenesis of both functional and organic gastrointestinal disorders. For example, SIBO/IMO may significantly contribute to irritable bowel syndrome (IBS), which affects up to 15% of the population [7-9]. A meta-analysis of 50 studies found that IBS patients were five times more likely to test positive for SIBO compared with healthy controls [10]. SIBO/IMO is also associated with inflammatory bowel disease [11,12]. Furthermore, recent literature has identified other associated conditions that may benefit from SIBO/IMO eradication therapy, including rosacea [13], restless leg syndrome [14,15], hepatic encephalopathy [16-18], non-responsive celiac disease [19], and metabolic abnormalities involving blood glucose, cholesterol, and potentially, body weight [20-22].

Clinicians have historically used the non-absorbable antibiotic, rifaximin, as the first-line therapy for most SIBO/IMO cases. Two meta-analyses reported that rifaximin eliminated SIBO for 59-67% of patients [23,24]. However, its high cost has limited its use in clinical settings. As many patients with IBS, SIBO/IMO, and other gastrointestinal disorders seek complementary and alternative therapies [25], researchers and clinicians have increasingly explored herbal antimicrobials as a treatment option for SIBO/IMO [26]. Although fewer studies have evaluated herbal antimicrobials compared to rifaximin, small-scale investigations suggest they may offer similar efficacy [27,28]. Research has also suggested that herbal antimicrobials may provide broader clinical benefits, including antidepressant and anti-inflammatory effects [29,30]. Their broader spectrum of activity may also help correct non-bacterial dysbiosis, such as overgrowths of fungal, parasitic, and protozoan microorganisms [31], while offering a potentially better side effect profile [27]. Thus, the use of herbal antimicrobial therapy has emerged as a viable alternative or adjunct to rifaximin for treating SIBO/IMO. 

A current challenge in treating SIBO/IMO involves the presence of biofilms - structured communities of microbial cells embedded in an extracellular polymeric substance matrix composed primarily of polysaccharides [32]. Bacteria that form biofilms can contribute to human disease and show increased resistance to antimicrobial therapies [33]. Researchers have linked biofilms to various gastrointestinal conditions, including *Helicobacter pylori *infection, Barrett's esophagus, and inflammatory bowel disease (IBD) [34].

Untreated biofilms and their associated microbial communities may act as a nidus for inflammatory responses within the gastrointestinal tract. They also represent a key mechanism underlying both antibiotic resistance and the persistence of dysbiosis [35]. In a study involving endoscopic examination of over 1,400 patients, Baumgartner et al. identified distinct biofilms in 57% of individuals with IBS and 34% of those with IBD, compared with only 6% of healthy controls [36]. 

In a set of guidelines published in 2014, the European Society of Clinical Microbiology and Infectious Diseases urged for further research into biofilm-targeted therapies for gastrointestinal diseases [37]. Of the proposed interventions, agents containing biofilm-degrading enzymes and chelators - intended to disrupt the matrix and release the embedded bacteria - were identified as particularly promising [37]. One such compound is N-acetylcysteine (NAC), a naturally occurring agent with the potential to inhibit and disrupt biofilm formation.

In an open-label, randomized controlled trial, investigators administered 600 mg of NAC prior to standard antimicrobial therapy in patients with treatment-resistant *H. pylori*. They reported eradication in 13 out of 20 patients in the NAC group versus four out of 20 in the placebo group (P < 0.010), attributing persistent infection largely to biofilm resistance rather than bacterial genotype or antimicrobial regimens [38]. 

Similarly, another randomized controlled trial found that adding NAC to a triple therapy regimen (amoxicillin/clarithromycin/omeprazole) improved *H. pylori *eradication rates compared to triple therapy alone (P = 0.005) [39]. However, a separate double-blind, randomized clinical trial found no significant benefit from adding NAC to a triple therapy regimen [40]. 

Although N-acetylcysteine is more widely studied for its anti-biofilm properties, enzyme-based agents are frequently used in clinical practice and have shown anecdotal efficacy in treating gastrointestinal symptoms. 

In this study, we retrospectively evaluated whether herbal antimicrobials, with or without adjunctive anti-biofilm agents, were associated with changes in breath test gas levels and the eradication of SIBO/IMO, as defined by modified North American Consensus Guidelines [41]. Additionally, we sought to compare the gas-level reductions associated with each type of anti-biofilm agent. Based on promising results from prior studies targeting bacterial biofilms, we hypothesized that adding anti-biofilm agents would be associated with greater gas-level reductions than herbal antimicrobials alone in treating SIBO/IMO.

## Materials and methods

This study is a retrospective chart review of patients who were previously randomized in a clinical protocol to receive herbal antimicrobials alone or herbal antimicrobials with adjunctive anti-biofilm agents for the treatment of SIBO or IMO. We identified 13 patients who met the following inclusion criteria: 1) a confirmed positive lactulose breath test (LBT) indicating SIBO or IMO; 2) completion of a prescribed 8-week treatment protocol; and 3) a follow-up LBT post-treatment. 

We extracted outcomes and additional variables from medical records at a single clinical site between April 2014 and July 2016. Although the original group assignment was randomized, this retrospective analysis is exploratory in nature and subject to limitations inherent to post hoc review, including missing data and selection bias. Comparisons between treatment arms may reflect associations but do not support definitive causal conclusions.

We applied the North American Consensus guidelines to determine positive results, defined as a hydrogen increase of >20 ppm at 90 minutes and/or a methane increase of >10 ppm at any point during the test [41]. Due to laboratory reporting constraints, we interpreted the rise in baseline hydrogen at 100 minutes rather than the North American Consensus guidelines of the 90-minute mark. Hydrogen SIBO eradication was defined per North American Consensus guidelines as a rise ≤20 ppm from baseline at 100 minutes. IMO eradication was defined as methane ≤10 ppm at all time points [41]. On average, follow-up breath testing occurred 125 days after the initial LBT. 

All 13 patients received a two-month herbal antimicrobial protocol. During the first month, each patient took Biota-Clear 1a (4 capsules twice daily) and Biota-Clear 1b (2 capsules twice daily), followed by Biota-Clear 2a (3 capsules twice daily) and Biota-Clear 2b (3 capsules twice daily) during the second month (Functional Medicine Formulations, Ropesville, USA). Table 1 lists the ingredients in each formula. In addition, eight patients received either N-acetyl cysteine (Designs for Health, Inc., Palm Coast, USA; 1 capsule twice daily away from meals) or Biota Dissolve (Functional Medicine Formulations, Ropesville, USA; 3 capsules twice daily away from meals) for eight weeks. Patients with prior *H. pylori *(n = 2) received NAC, whereas those without (n = 6) received Biota Dissolve. Table 2 lists the ingredients in each formula. NAC was selected for those with a prior *H. pylori *history based on evidence that it can enhance bacterial eradication [38]. 

**Table 1:** Herbal antimicrobial ingredients *Dose provided for 1 capsule

Formula	Ingredients*	Daily Dose
Biota-Clear 1a	Oregano oil (*Origanum vulgare*) (leaf) (60 mg)	4 capsules twice daily
Biota-Clear 1b	Tribulus extract (*Tribulus terrestris*) (whole herb) (200 mg)	2 capsules twice daily
	Magnesium caprylate (150 mg)	
	Phellodendron (*Phellodendron amurense*) (bark) (100 mg)	
	Black walnut powder (*Juglans nigra*) (hull) (100 mg)	
	Barberry extract (*Berberis vulgaris*) (root) (50 mg)	
	Artemisinin (from sweet wormwood) (*Artemisia annua*) (whole plant) (15 mg)	
	Bearberry extract (*Arctostaphylos uva-ursi*) (leaf) (100 mg)	
Biota-Clear 2a	Wormwood (*Artemisia absinthium*) (whole plant) (150 mg)	3 capsules twice daily
	Olive (*Olea europaea*) (leaf) (100 mg)	
	Black walnut (*Juglans nigra*) (Hull) (85 mg)	
	Berberine hydrochloride (75 mg)	
	Artemisinin (25 mg)	
Biota-Clear 2b	Biotin (1,000 mcg)	3 capsules twice daily
	Sodium (As sodium caprylate) (12.5 mg)	
	Pau d’arco (*Tabebuia heptaphylla*) (bark) (150 mg)	
	Black walnut (*Juglans nigra*) (hull) (100 mg)	
	Sodium caprylate (100 mg)	
	Oregano (*Origanum vulgare*) (leaf) (100 mg)	

**Table 2:** Anti-biofilm agent ingredients *Dose provided for 1 capsule EDTA: ethylenediaminetetraacetic acid; DPP-IV: dipeptidyl peptidase 4

Formula	Ingredients*	Daily Dose
N-Acetylcysteine	N-Acetylcysteine (900 mg)	1 capsule twice daily
Biota-Dissolve	Sodium (as disodium EDTA) (15 mg)	3 capsules twice daily
	Proprietary Enzyme/EDTA Blend (337.5 mg), providing the following:	
	Glucoamylase (with isomaltase side chain activity)	
	Chitosanase	
	Cellulase	
	Hemicellulase/pectinase complex	
	Beta-glucanase	
	Protease/peptidase complex with endopeptidase, exopeptidase, and DPP-IV activity	
	Lysozyme (from egg white)	
	Serratia peptidase (enteric-coated)	
	EDTA (as disodium EDTA)	

We conducted statistical analyses using IBM SPSS Statistics Version 29 (IBM Corp., Armonk, USA). Independent-samples t-tests were used to compare mean changes in hydrogen and methane levels between the treatment and control groups. Paired-samples t-tests were used to assess pre- to post-treatment changes within each group. A P value of <0.05 was considered statistically significant.

## Results

Baseline characteristics

This retrospective chart review included 13 patients: five in the control group (herbal antimicrobials only) and eight in the treatment group (herbal antimicrobials with adjunctive anti-biofilm agents). Two patients in the treatment group had a history of *H. pylori*; the other six did not. Patient ages ranged from 26-52 years. The mean age did not differ significantly between groups (control: M = 41.20 years; treatment: M = 40.50 years; P = 0.889). The control group included four females and one male, while the treatment group included seven females and one male. The treatment group included seven out of the 11 total women (64%) and one out of the two men (50%). These gender distributions did not differ significantly (P = 0.715) (Table 3).

**Table 3:** Baseline characteristics of the treatment and control groups Hydrogen (H_2_) and methane (CH_4_) measured in parts per million (ppm). Abbreviations: SIBO - small intestinal bacterial overgrowth; IMO - intestinal methanogen overgrowth *No significant difference between groups; **Measured at 100 minutes during lactulose breath test (LBT); ***Highest observed level during LBT

Variable	Control* (n = 5)	Treatment* (n = 8)
Age, mean years	41.2	40.5
Female, n (%)	4 (80)	7 (87.5)
Male, n (%)	1 (20)	1 (12.5)
Pre-Test H_2_**	20.80	51.00
Pre-Test CH_4_***	42.00	54.38
H_2_ SIBO, n (%)	2 (40)	5 (62.5)
CH_4_ SIBO, n (%)	4 (80)	6 (75)

Time between pre-tests and post-tests

The interval between pre-treatment and post-treatment breath tests ranged from 56 to 154 days. The mean duration was 125.15 days (SD = 17.748). The treatment group had a slightly longer average interval (M = 129.13 days) than the control group (M = 118.80); however, the approximately 10-day difference was not statistically significant (t = -1.022, df = 11, P = 0.329).

Change in hydrogen and methane gas

The treatment group showed a higher mean pre-test hydrogen level (M = 51.00 ppm) than the control group (M = 20.80 ppm). To assess whether baseline differences might have influenced the outcomes, we performed independent samples t-tests. The difference in pre-test hydrogen levels was not statistically significant (P = 0.087). Pre-test methane levels also did not differ significantly between groups (Table 3).

**Figure 1:**
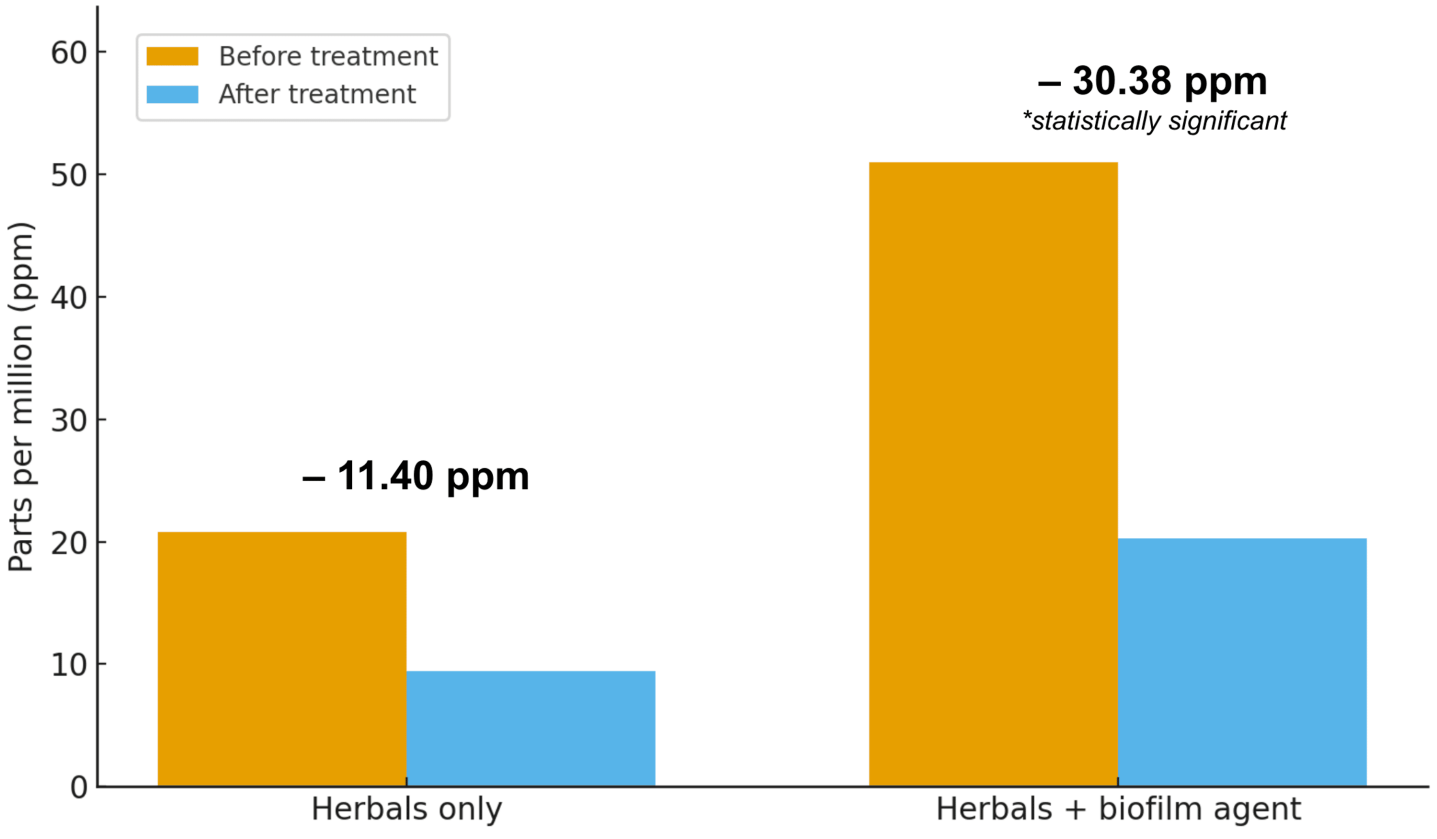
Pre and post-treatment hydrogen levels in the control and treatment groups

We conducted paired-samples t-tests to evaluate within-group changes in hydrogen and methane levels from pre-test to post-test. In the control group, hydrogen levels decreased (M = -11.40 ppm), but this reduction was not statistically significant (t = -2.066, df = 4, P = 0.108). In contrast, the treatment group showed a significant reduction in hydrogen, with mean levels dropping from 51.00 ppm to 20.25 ppm (t = -3.742, df = 7, P = .007) (Table 4, Figure 1). 

**Table 4:** Pre- and post-treatment hydrogen and methane levels in the control and treatment groups Hydrogen (H_2_) and methane (CH_4_) measured in parts per million (ppm). *Measured at 100 minutes during lactulose breath test (LBT); **Highest observed level during LBT

	Control (n = 5)	Treatment (n = 8)
Hydrogen (H_2_)		
Pre-test H_2_*	20.80	51.00
Post-test H_2_*	9.40	20.25
H_2_ change	-11.40	-30.75
P value	0.108	0.007
Methane (CH_4_)		
Pre-test CH_4_**	42.00	54.38
Post-test CH_4_**	40.00	28.00
CH_4_ change	-2.00	-26.38
P value	0.849	0.042

**Figure 2:**
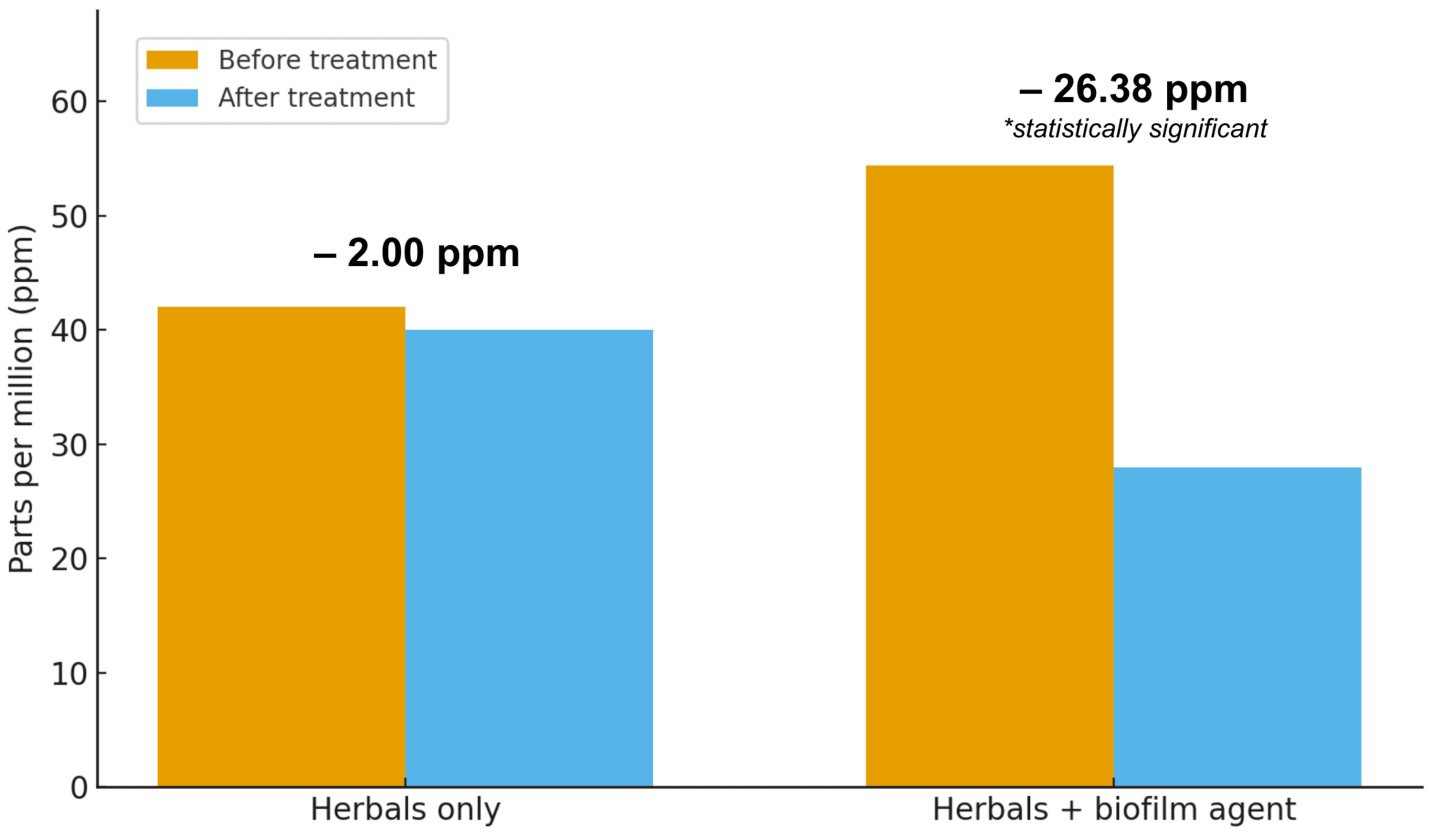
Pre and post-treatment methane levels in the control and treatment groups

Methane levels in the control group declined from 42.00 ppm to 40.00 ppm (t = -0.212, df = 4, P = 0.849). The treatment group demonstrated a larger and statistically significant reduction in methane, from 54.38 to 28.00 ppm (t = 2.480, df = 7, P = 0.042) (Table 4, Figure 2). 

We also analyzed gas level reductions across the full sample. Hydrogen levels significantly decreased from 39.39 ppm to 16.08 ppm (t = -3.910, df = 12, P = 0.002). Although methane levels dropped from 49.62 ppm to 32.69 ppm, this reduction was not statistically significant (t = -1.981, df = 12, P = 0.071).

SIBO and IMO eradication

Follow-up lactulose breath testing showed that 100% of patients in the control group and 60% in the treatment group no longer met criteria for SIBO after the intervention. However, this difference was not statistically significant (P = 0.928). 

We also compared differences in eradication rates for SIBO within the treatment group, control group, and overall cohort. SIBO eradication rates were 60% in the treatment group, 100% in the control group, and 71.5% overall, with no significant differences between groups (Table 5). In contrast, the eradication rate for IMO was 0% across all groups (Table 6). 

**Table 5:** Hydrogen breath test (H2) positivity before and after treatment by group Abbreviation: H_2_ = Hydrogen

Group	Pre-Test H_2 _Positivity n (%)	Post-Test H_2_ Positivity n (%)	Change in H_2_ Positivity n (%)	P
Treatment	5 (100%)	2 (40%)	-3 (-60%)	0.096
Control	2 (100%)	0 (0.0)	-2 (-100%)	1.00
Overall cohort	7 (100%)	2 (28.5%)	-5 (71.5%)	0.104

**Table 6:** Methane breath test positivity (CH4) before and after treatment by group Abbreviation: CH_4_ = Methane

Group	Pre-Test CH_4_ Positivity n (%)	Post-Test CH_4_ Positivity n (%)	Change in CH_4_ Positivity n (%)	P
Treatment	6 (100%)	6 (100%)	0 (0.0)	1.00
Control	4 (100%)	4 (100%)	0 (0.0)	1.00
Overall cohort	10 (100%)	10 (100%)	0 (0.0)	1.00

Differences between biofilm disruptor types

Within the treatment group, we explored whether gas reductions differed by the type of biofilm disruptor. Patients with a history of *H. pylori *received N-acetylcysteine (NAC) (n = 2), while the remaining patients (n = 6) received an enzyme-based blend (Biota Dissolve). 

The mean reduction in hydrogen levels did not differ notably between groups (t = -0.653, df = 5.929, P = 0.704). For methane, the enzyme group showed a greater mean reduction (-35.17 ppm) than the NAC group (-6.50 ppm), with a P value of 0.046. However, this comparison was exploratory and limited by the small sample size and non-random group assignment.

## Discussion

**Figure 3:**
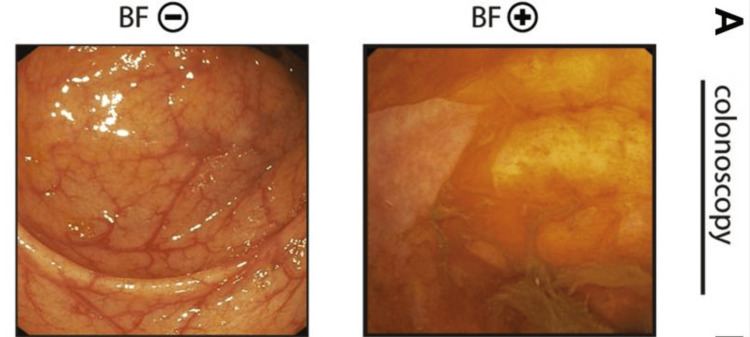
Endoscopic visualization of biofilm negative (BF⁻) and biofilm positive (BF⁺) patients Adapted from Baumgartner M, Lang M, Holley H, et al.: Mucosal biofilms are an endoscopic feature of irritable bowel syndrome and ulcerative colitis. Gastroenterol. 2021, 161:1245-56.e20. 10.1053/j.gastro.2021.06.024 [36]. Licensed under Creative Commons CC BY 4.0.

This retrospective study assessed changes in hydrogen and methane levels in patients with SIBO and IMO following treatment with herbal antimicrobial therapy, with or without adjunctive biofilm disruptors. We found that combining anti-biofilm agents with herbal antimicrobials resulted in larger reductions in hydrogen (−30.75 vs −11.40 ppm, P = 0.007) and methane levels (−26.38 vs −2.00 ppm, P = 0.042) compared with herbal antimicrobials alone. Biofilms are dense microbial communities embedded in an extracellular matrix that can protect microbes from antimicrobial agents, contributing to the chronicity and treatment resistance of gastrointestinal conditions such as IBS, IBD, and *H. pylori *[34,36]. Supporting this, Baumgartner et al. examined more than 1,400 patients and identified endoscopically visible biofilms in 57% of those with IBS, 34% with IBD, and 6% in healthy controls (Figure 3) [36], highlighting their prevalence and potential role in shielding microbes from therapy. The greater reductions in hydrogen and methane gas observed in our patients receiving adjunctive anti-biofilm therapy may reflect disruption of this matrix.

SIBO eradication rates, defined by modified North American Consensus criteria, were 60% in the treatment group and 100% in the control group. These rates did not differ significantly and may have been influenced by the small sample size and retrospective design. IMO eradication was not observed in either group; however, the treatment group experienced significantly greater reductions in methane levels (-26.38 ppm) compared to the control group (-2.00 ppm). This suggests that, although complete eradication was not achieved, adjunctive use of biofilm disruptors may be associated with a more pronounced reduction in methane production, warranting further investigation into extended or modified treatment protocols for IMO. Symptom data were not available in this analysis, so it remains unknown whether the observed reductions in hydrogen and methane levels translated to clinical improvement. To our knowledge, this is the first study to assess the efficacy of anti-biofilm agents in combination with herbal antimicrobials for the treatment of SIBO or IMO, and among the few studies to examine herbal antimicrobial effects using LBT-based outcomes for these conditions [27,42]. Rifaximin has been reported to achieve SIBO eradication rates of 59-67% [23,24]. Our study demonstrated a comparable rate of 60-100% using herbal antimicrobials, consistent with or slightly higher than those reported in similar studies [27,42]. Within the treatment group, patients who received the anti-biofilm enzyme blend experienced a greater mean reduction in methane levels than those who received N-acetylcysteine (NAC). Specifically, the mean reduction in methane was 35.17 ppm in the enzyme group and 6.50 ppm in the NAC group. However, this subgroup analysis was not randomized, and the NAC group consisted of only two patients. Moreover, the baseline methane levels were notably higher in the enzyme group (67.17 ppm) than in the NAC group (16.00 ppm), further limiting direct comparisons. 

Previous studies have proposed that anti-biofilm agents may support antimicrobial therapy by disrupting the extracellular polymeric substance matrix, inhibiting microbial adhesion, reducing virulence factor production, and interfering with quorum sensing [43,44]. The observed reductions in gas levels in this study are consistent with these proposed mechanisms and support further evaluation of anti-biofilm strategies in SIBO/IMO treatment. Although overall eradication rates were not statistically significant, the enhanced gas reductions in the treatment group suggest a potentially augmented treatment response when biofilm disruptors are used alongside herbal antimicrobials.

Limitations and future research

This study has several limitations inherent to its retrospective design. The time interval between the pre- and post-treatment breath tests was not standardized, ranging from 56 to 154 days, with a mean of 125 days. This uncontrolled variation in follow-up timing may have impacted the observed results. Patients with longer gaps between tests may have experienced changes in bacterial overgrowth unrelated to the intervention. The treatment group, on average, had a 10-day longer interval than the control group, though this difference was not statistically significant. 

Furthermore, the study did not assess symptom improvement, and as a result, it cannot draw conclusions about clinical outcomes or patient-reported benefits. Although some studies report a correlation between breath test gas levels and symptom relief [45-47], others do not support this association [48-51]. For example, in one study, 78 patients with diarrhea-predominant irritable bowel syndrome (IBS-D) reported symptom improvement after rifaximin treatment, even though only 45% had normalization of breath test results [48]. 

The two main carbohydrate substrates used for SIBO/IMO breath testing are glucose and lactulose. Our study employed lactulose, though debate remains over which substrate is most clinically informative. For instance, one meta-analysis suggests that glucose breath testing may provide greater sensitivity and specificity for detecting SIBO [52]. In contrast, a more recent study of 6,000 participants reported that lactulose testing was more frequently positive and more closely associated with symptoms than glucose [53]. Therefore, the choice of substrate could have influenced both diagnostic accuracy and the assessment of treatment outcomes.

The small sample size (N = 13) in this study limits the generalizability of our findings. Despite the limited cohort, the analysis still identified statistically significant differences at the P ≤ 0.05 level. However, the small sample increases the risk of both type I and type II errors. This study was underpowered, and some findings may reflect false positives and require replication in larger cohorts. 

To build on this study’s findings, future studies should employ randomized, placebo-controlled clinical trial designs to reduce bias and improve the strength of the evidence. Additional research is needed to evaluate the comparative effectiveness of herbal antimicrobials, to clarify the role of biofilm disruptors, and to determine whether reductions in gas levels translate into meaningful clinical improvements. Although many anti-biofilm agents are generally regarded as safe [54], concerns regarding cost, tolerability, and practical application remain [55-57]. These factors may limit their routine use until additional evidence clarifies their benefit and optimal application in SIBO/IMO treatment. As SIBO/IMO treatment remains partially empirical due to limited high-quality evidence, more rigorous trials are essential to guide evidence-based therapeutic decisions.

Potential clinical implications

While we acknowledge these insights are speculative and based on a limited evidence base, clinical decisions must often be guided by the best available data. 

Both rifaximin and herbal antimicrobials appear to be reasonable first-line options for hydrogen-dominant SIBO, with comparable eradication rates. The choice of therapy can be individualized based on factors such as cost, availability, prior treatment response, and patient preference. If improvement is limited after one month, adding a biofilm-disrupting agent containing enzymes and ethylenediaminetetraacetic acid (EDTA)may enhance treatment efficacy, particularly in cases where prior antimicrobial monotherapy has failed.

For intestinal methanogen overgrowth (IMO), our findings - along with emerging data [58] and clinical experience - suggest that longer treatment durations may be necessary. In our study, methane levels were significantly reduced by month 2, yet no patients met criteria for full eradication. Similarly, another study [58] using herbal antimicrobials observed 0% eradication at week 10, rising to 25% by week 14. These findings support the hypothesis that IMO responds more slowly and may require a more aggressive or sustained approach.

Given this, we recommend initiating IMO treatment with both an antimicrobial (herbal or pharmaceutical) and an enzyme-EDTA-based biofilm disruptor from the outset, and considering extending therapy beyond the typical duration when clinically appropriate. In practice, setting patient expectations around the need for longer courses of treatment for IMO can also improve adherence and outcomes.

## Conclusions

In this retrospective study, the addition of anti-biofilm agents to herbal antimicrobials resulted in significant reductions in both hydrogen and methane levels. Herbal antimicrobials, with or without anti-biofilm therapy, achieved SIBO eradication rates of 60-100%; however, these differences were not statistically significant, likely due to the small sample size. While IMO eradication was not observed, the pronounced reduction in gas levels among patients receiving adjunctive enzyme-EDTA-based biofilm disruptor therapy suggests that longer treatment or follow-up may have yielded higher IMO eradication rates. This study contributes to the growing body of evidence supporting the use of herbal antimicrobials for managing SIBO/IMO and highlights the potential benefit of enzyme-EDTA-based biofilm disruptors as adjunctive agents. Future prospective, controlled studies are needed to confirm these findings and better define the role of biofilm disruptors in SIBO/IMO management.
